# Ghana’s Livelihood Empowerment Against Poverty (1000) Program Seasonally Impacts Birthweight: A Difference-in-Differences Analysis

**DOI:** 10.3389/ijph.2023.1605336

**Published:** 2023-02-20

**Authors:** Sarah Quinones, Pauline Mendola, Lili Tian, Shao Lin, Jacob Novignon, Gustavo Angeles, Tia Palermo

**Affiliations:** ^1^ Department of Epidemiology and Environmental Health, School of Public Health and Health Professions, University at Buffalo, State University of New York, Buffalo, NY, United States; ^2^ Department of Biostatistics, University at Buffalo, State University of New York, Buffalo, NY, United States; ^3^ Department of Environmental Health Sciences, University at Albany, State University of New York, Rensselaer, NY, United States; ^4^ Department of Epidemiology and Biostatistics, University at Albany, State University of New York, Rensselaer, NY, United States; ^5^ Department of Economics, Kwame Nkrumah University of Science and Technology, Kumasi, Ghana; ^6^ Department of Maternal and Child Health, Gillings School of Global Public Health, University of North Carolina at Chapel Hill, Chapel Hill, NC, United States

**Keywords:** Ghana, cash transfers, LEAP 1000, birthweight, LBW

## Abstract

**Objectives:** Low birthweight (LBW) prevalence remains high in African countries and evidence of cash transfer impacts on birthweight, particularly by season of infant birth, is limited. This study examines overall and seasonal cash transfer impacts on LBW in rural Ghana.

**Methods:** Data come from a longitudinal, quasi-experimental impact evaluation of the Livelihood Empowerment Against Poverty (LEAP) 1,000 unconditional cash transfer program for impoverished pregnant or lactating women in rural districts of Northern Ghana. LEAP1000 program impacts on average birthweight and LBW were estimated for a multiply imputed sample of 3,258 and a panel sample of 1,567 infants using differences-in-differences models and triple difference models to assess impacts by season.

**Results:** LEAP1000 decreased LBW prevalence by 3.5 and 4.1 percentage points overall and in the dry season, respectively. LEAP1000 increased average birthweight by 94, 109, and 79 g overall, in the dry season, and in the rainy season, respectively.

**Conclusion:** Our findings of positive LEAP1000 impacts on birthweight across seasons and on LBW in the dry season demonstrate the need to take seasonal vulnerabilities into account when designing and implementing programs for rural populations in Africa.

## Introduction

Birthweight is a salient indicator of infant and maternal health with short- and long-term health implications (1). Low birthweight (LBW, birthweight < 2.5 kg) is associated with increased risk of neonatal morbidity and mortality (2, 3), stunting (4), and reduced academic achievement (5). The etiology of LBW is complex; it is estimated that approximately 40% of LBW is hereditary while the other 60% environmental (6). Environmental factors including maternal nutrition, work, and psychosocial stress, smoking, alcohol, caffeine, drug consumption, and exposures to toxicants, pollution, and extreme increase LBW risk (7–10). Prevalence of LBW is highest in low- and middle-income countries (LMICs) of Africa and Southeast Asia (11). In sub-Saharan Africa (SSA), 14% of newborn infants were LBW and 12% were preterm in 2015, and rates of neonatal and maternal mortality are high in Ghana (23 per 1,000 and 308 per 100,000 live births, respectively) (11–13).

Multi-dimensional poverty is pervasive in Africa. About 59.6% of people suffer from some form of food insecurity compared to a global average of 30.4% (14). Also, 38.3% of the population in SSA are estimated to be living in poverty compared to a global average of 8.6% in 2018 (15). In response to this widespread poverty, many countries in Africa have implemented social protection programs to improve the health and wellbeing of impoverished and vulnerable populations (16).

Evidence indicates that social protection programs, which include cash transfers, reduce poverty (17), promote household food security (18), increase uptake of health insurance (19), and healthcare utilization (20–22). Socioeconomic status, food security, and healthcare utilization are associated with LBW, suggesting potential pathways through which these poverty-reduction programs might also impact birthweight (23). Despite a broader evidence base on impacts of these potential mediators of adverse birth outcomes, the impacts of cash transfers on birthweight are less studied (24).

The Republic of Ghana has a tropical climate characterized by varying temperature and precipitation patterns (25). In the south, maximum precipitation occurs between April to June and September to November. The north, however, experiences a single rainy season from March to October. During the rainy season, agricultural output is diminished and, subsequently, hunger and food insecurity increase in concert with increased agricultural labor and malaria transmission, which have been associated with increased risk of LBW (26).

In the current study, we aim to examine how an unconditional cash transfer targeted to impoverished pregnant and lactating women in rural households of Northern Ghana impacts birthweight and whether season of birth modifies these impacts, given dramatic differences in rainfall, labor, exposures, vector- and water-borne diseases and availability of food by season in this region.

### Program Description

The Livelihood Empowerment Against Poverty (LEAP) program, Ghana’s flagship social protection program, has been ongoing since 2008. The program was originally targeted to extremely poor households with an elderly person, person with disability, orphan, or vulnerable child. LEAP eligible households were initially selected using a community-based approach, then verified centrally using a proxy means test (PMT) (27). Specifically, data were collected from participants of a larger nationally representative sample surveyed as part of a research study conducted by the Institute for Statistical, Social, and Economic Research (ISSER) and Yale University in 2010. The evaluation sample of the LEAP program was comprised of nearly 6,000 households in 7 urban and rural districts across 3 regions of Ghana.

Starting in 2015, the LEAP1000 (a sub-component of the broader LEAP program) was piloted in 10 districts of two northern regions of Ghana which were selected based on high proportions of poverty in their populations and high incidence of poor nutrition. The pilot was targeted to extremely poor households with a pregnant or lactating woman (one eligible woman per household) using the same community-based targeting and central verification with PMT approaches as LEAP. Priority was given to communities that were not yet participating in LEAP. This expanded targeting approach was informed by the understanding that the first 1,000 days of life are critical to child development (28), including stunting (29). Eligibility criteria to apply to LEAP1000 was based on presentation of 1) antenatal cards (if pregnant); or 2) birth certificates and weighing cards among households with infants less than 15 months; and eligibility for enrollment was determined by a PMT score based on assets, dwelling characteristics, household size, and other characteristics reported by the target woman in the household (range: 6–9). In its pilot phase, the Ghana LEAP1000 program provided bi-monthly cash transfers (GHC₵ 64 to GHC₵ 106; equivalent to approximately 17–28 USD in October 2015 exchange rates) combined with premium fee waivers to enroll in the National Health Insurance Scheme (NHIS) to approximately 6,000 households in 10 districts of the Northern and Upper East regions of Ghana (30). These NHIS fee waivers in concert with the free maternal health policy implemented in 2008 allowing all pregnant women free NHIS enrollment mitigated the costs of seeking care during and after pregnancy (31). A primary objective of the LEAP1000 program was to reduce stunting among this population. Thus, we conducted this secondary analysis of LEAP1000 data to examine LEAP1000 impacts on LBW, an antecedent to stunting.

We hypothesize that the Ghana LEAP1000 program leads to reductions in LBW and increases in average birthweight and these impacts will be larger among infants born in the dry season compared to the rainy season, as the dry season is characterized by hotter temperatures and fields ready for harvest, thus requiring pregnant women to tend to crops in less favorable outdoor conditions in the end stages of pregnancy, when growth rates of the fetus is most rapid and subject to perturbation. This study contributes to a dearth of evidence on cash transfer impacts on birthweight and is the first to examine whether these impacts vary by season of birth. With this information, design and implementation of cash transfer programs may be better informed to protect pregnant women and their infants at the most vulnerable periods of development.

## Methods

### Study Design

**FIGURE 1:**
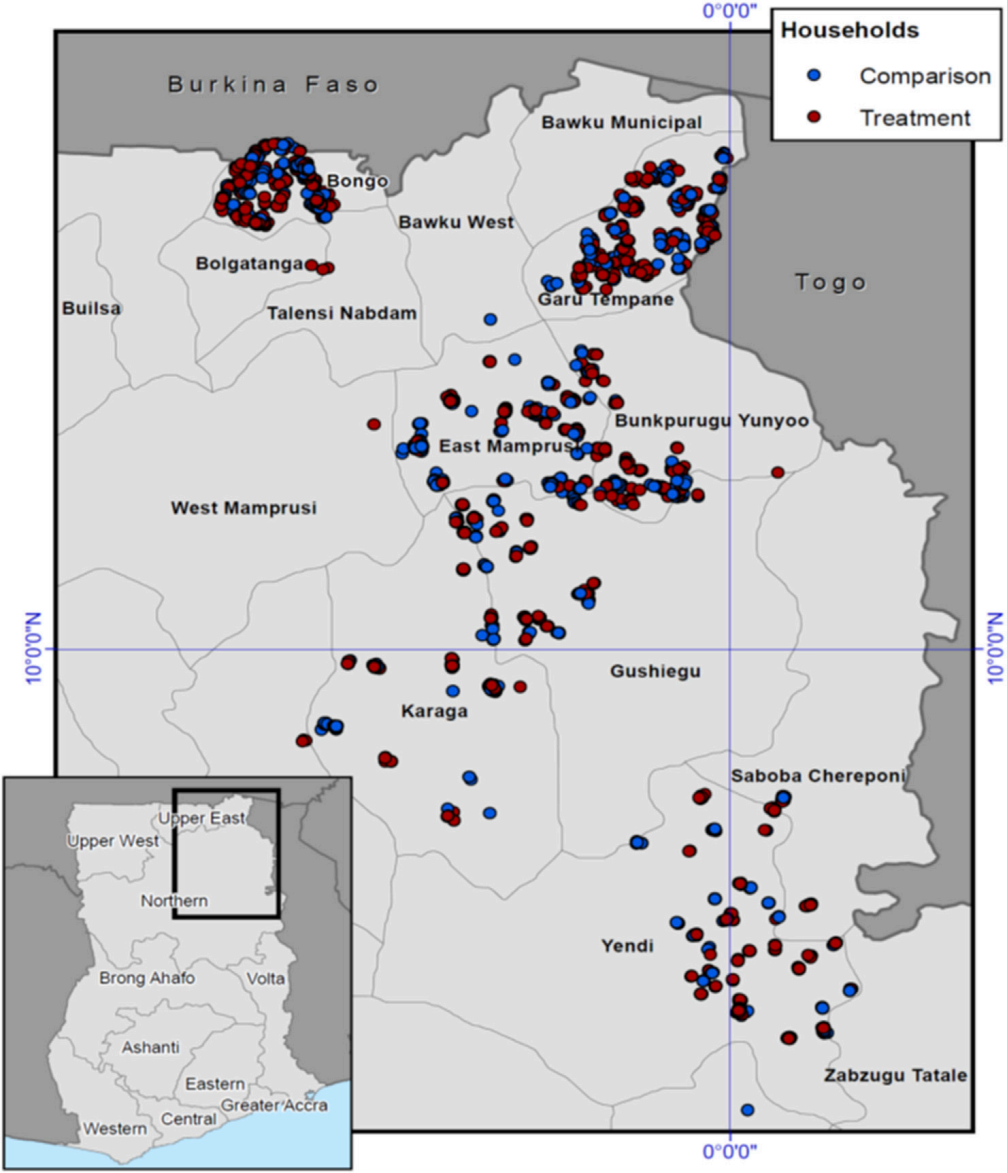
Map of 5 selected districts and the distribution of treatment and comparison households in Northern Ghana, 2015–2017.

This study uses data from the baseline (July–September 2015) and endline (June–August 2017) surveys of the Ghana LEAP1000 impact evaluation conducted by UNICEF’s Office of Research—Innocenti, the University of North Carolina at Chapel Hill, and the Institute of Statistical, Social and Economic Research (ISSER) at the University of Ghana. Due to budget restrictions, 5 districts were selected for the impact evaluation. A map of study districts and households is presented in Figure 1. Households were targeted for enrollment into LEAP1000 between March and July 2015. Baseline surveys were conducted prior to cash transfer roll-out, and the first payments occurred at the end of September 2015.

The quasi-experimental study design utilizes a sampling strategy inspired by a regression discontinuity design (RDD) that exploits an eligibility threshold based on the PMT cut-off to sample a valid comparison group. Households were systematically sampled around the cut-off (comparison and treatment households were within 0.46 SD and 0.43 SD, respectively). Treatment and comparison households were drawn from the same communities. A total of 8,058 households from 189 different communities applied to LEAP1000 in the five districts used in the impact evaluation. Based on the PMT cut-off score, 3,619 households qualified for LEAP1000 enrollment, and 4,439 households did not qualify. Based on power calculations, 1,250 households (+10% reserve sample to be used for replacement) falling immediately on either side of the cut-off were targeted for the impact evaluation (the sample also used for this study). A complete sample of 1,235 comparison households and 1,262 LEAP1000 beneficiary households were successfully interviewed for the impact evaluation. More details on the methodology of the Ghana LEAP1000 sampling approach and analyses used for the impact evaluation can be found elsewhere (32–36).

### Sample and Data Collection

At baseline, 2,497 household interviews were conducted and 2,331 (93.4%) of those households were re-interviewed at endline. Respondents to the household interviews were the female primary beneficiary of LEAP1000. Questionnaires were administered in a private location in the household or yard by female enumerators given the sensitive nature of some of the questionnaire topics. Responses were input into a tablet using CSPro software. Questionnaire topics included household composition, household members’ education and health, water, sanitation, and hygiene, food security, time use and livelihood activities, reproductive health (women aged 12–49 years), birth history, maternal and newborn health, immunizations, among others. The LEAP1000 eligible household member answered questions relating to birth history in the past 3 years at baseline and since baseline (at endline).

### Measures

The primary outcomes for this study include LBW as a dichotomous variable (<2.5 kg) and continuous birthweight for live births (0–36 months old at baseline or born since baseline at endline) observed on infant health cards or reported by mothers (when health card unavailable). Birthweight is normally distributed in this sample. LEAP1000 program participation (treatment) was the main independent variable of interest. Other variables controlled for included PMT score, household size, female household head, district of residence [Karaga, Bongo, Garu Tempane, Yendi (referent: East Mamprusi)], infant month and year of birth, and number of household members aged 0–17 years old. A dummy variable for birth during the rainy season was equal to 1 if the infant was born between March and October and equal to 0 otherwise.

### Statistical Analysis

To estimate the impact of LEAP1000 on birthweight, we used a difference-in-difference (DID) approach. DID compares the changes in the outcome (i.e., birthweight and LBW) of the treatment group between baseline and endline to changes in the outcome in the comparison group over the same period, and any difference between these differences is attributed to the program. The equation is specified as follows:

$$Y_{ijt}=\beta_{0}+\beta_{1}P_{ij}+\beta_{2}T_{t}+\beta_{3}P_{ij}*T_{t}+{X_{ij,0}\beta }_{4}+\varepsilon_{ijt}$$ (1)

where $Y_{ijt}$ is continuous birthweight and dichotomous LBW for child i who lives in community (village) j at time t. $P_{ij}$ is a binary variable that represents LEAP1000 program participation (equal to 1 if the household to which i belongs in community j is enrolled, and equal to 0 if not). *T*_*t*_ is a binary indicator for time of the observation (equal to 1 if in the endline survey, and equal to 0 if in baseline survey). $P_{ij}∙T_{t}$ is the interaction of the program treatment variable and the time variable and $\beta_{3}$ indicates the treatment effect. X represents a set of observed individual and household characteristics described above, measured at baseline. *ε*_*ijt*_ is the normally distributed random error term.

A key assumption of the DID model is the “parallel trends assumption” which assumes that the intervention group would have experienced changes in outcomes similar to those of the control group in the absence of the program. Treatment and comparison households were sampled from the same communities, meaning they were exposed to similar circumstances (e.g., weather patterns, inflation, etc.) before and during program implementation. Baseline balance tests are used to test this assumption and variables that differ between treatment and comparison groups at baseline are adjusted for in regression models to maintain the comparability of these groups.

Next, we test whether program impacts were moderated by season of birth using a triple difference approach specified as follows:

$$Y_{ijt}=\beta_{0}+\beta_{1}P_{ij}+\beta_{2}T_{t}+\beta_{3}P_{ij}T_{t}+\beta_{4}{Rainy}_{ij}+\beta_{5}P_{ij}{Rainy}_{ij}+\beta_{6}T_{t}{Rainy}_{ij}+\beta_{7}P_{ij}T_{t}{Rainy}_{ij}+{X\beta }_{8ij,0}+\varepsilon_{ijt}$$ (2)

where all equation parameters are the same as those in Eq. 1 except the triple difference model (DDD) includes a binary indicator for birth in the rainy (vs. dry) season for child *i* in community *j* (Rainy) and this term is interacted with treatment and time and the $P_{ij}∙T_{t}$ interaction. For infants born in the dry season, the program impact is indicated by $\beta_{3}$ *.* The program impact for infants born in the rainy season is indicated by ${\beta_{3}+\beta }_{7}$ , and we estimated the joint significance of these coefficients ( ${\beta_{3}+\beta }_{7}$ ) with 95% confidence intervals (CIs) and two-sided *p*-values using the *lincom* command in Stata. All models adjust for clustering of standard errors at the community level.

### Multiple Imputation (MI)

**FIGURE 2:**
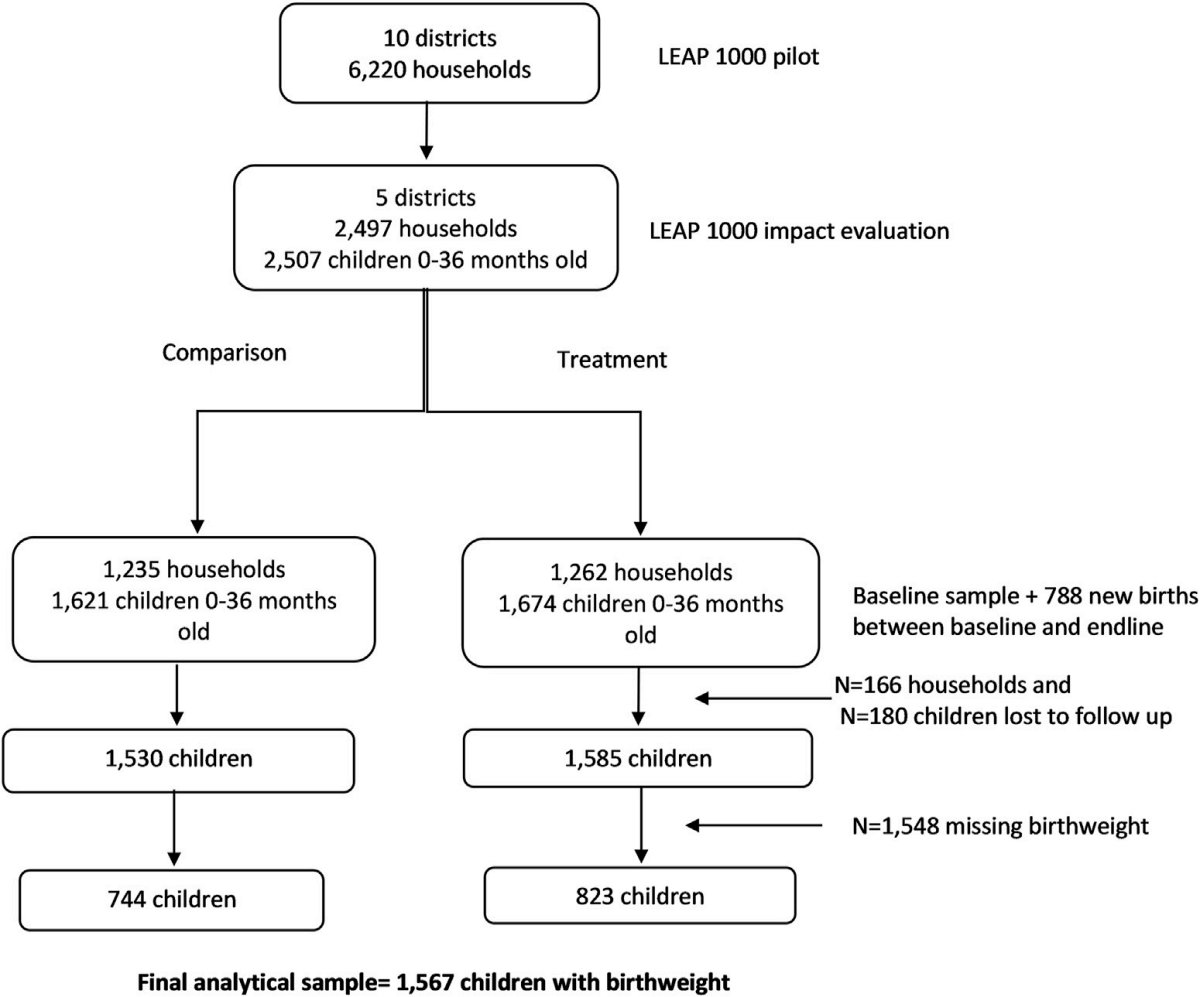
CONSORT diagram for LEAP1000 impact evaluation on birthweight. Livelihood Empowerment Against Poverty in the first 1,000 days of life impact evaluation, Ghana, 2015–2017.

Figure 2 shows that our sample is missing 50% of the information on birthweight. To explore the potential bias in this approach, we apply chained MI (MICE) to examine these pooled and season-specific associations for all children 0–36 months old living in LEAP1000 households at baseline and endline. Using MICE, we imputed birthweight using all dependent variables and covariates specified in Eqs 1, 2 in addition to auxiliary variables that were strongly correlated (r > 0.40) with missingness of birthweight including infant age in months and delivery in a health facility. This imputed birthweight was then used to generate the dichotomous LBW variable.

### Sensitivity Analyses

Given that birthweight in this context has been shown to be heaped (37–39) (or grouped at 100- or 500-g intervals), we created another LBW variable inclusive of the 2.5 kg cut-off. Further, the construction of our season variable is rather rigid and may lead to spurious conclusions as an infant can be born in November, which is technically the dry season, however, most of their gestation occurred during the rainy season. To address this, we run a DDD model interacting program impacts with the number of months of *in utero* rainy season exposure in the last 3 months of pregnancy.

### Ethics Statement

The original evaluation study was reviewed by the Ethics Committee for the Humanities of the University of Ghana. The trial is registered in the International Initiative for Impact Evaluation’s (3ie) Registry for International Development Impact Evaluations (BLINDED) and the Pan African Clinical Trial Registry (BLINDED). The current analysis uses de-identified data and was thus exempted from IRB review at the BLINDED. All participating individuals provided informed consent.

## Results

The baseline sample included 2,497 households and at endline 2,331 households were re-interviewed (93.4% follow-up rate). The final analytic sample for this study includes 1,567 infants with birthweight information from the panel sample of 2,331 households interviewed at baseline (1,212 births) and endline (355 births; Figure 2).

The maternal-, infant-, and household-level baseline sample characteristics are presented in Table 1 by treatment status. We achieved baseline balance of our primary birthweight outcomes, meaning there are no significant differences between groups in these measures (Table 1). Monthly averages of birthweight do not differ significantly between treatment and comparison groups at baseline or endline (Supplementary Figure S1). Given the sampling criteria, the mean PMT score of the comparison group was higher than that of the treatment group (*p* < 0.001). Further, the treatment group had a larger average household size (*p* < 0.001), older household heads (*p* = 0.010), more female-headed households (*p* = 0.077), and less household heads with formal education compared to the comparison group (*p* = 0.007). Treatment women sought ANC from a skilled provider more than comparison women (*p* = 0.009). A higher percentage of comparison women lived in East Mamprusi compared to treatment women (*p* = 0.023). The imbalanced variables at baseline served as covariates in regression models.

**TABLE 1:** Maternal-, infant-, and household-level characteristics at baseline, by treatment status (N = 1,327). Livelihood empowerment against poverty in the first 1,000 days of life impact evaluation, Ghana, 2015–2017.

	N (%) or mean [95% CI]		
	Comparison	Treatment	*p*-value^a^
Maternal-level			
PMT score	7.23 [7.22, 7.23]	7.09 [7.08, 7.09]	<0.001
ANC from skilled provider	624 (98.3)	690 (99.7)	0.009
ANC 4 times or more	572 (90.1)	636 (91.9)	0.224
Delivery with assistance from skilled provider	593 (93.4)	639 (92.3)	0.461
Delivery in health facility	590 (92.9)	636 (91.9)	0.490
Infant-level			
Age (months)	10.6 [9.91, 11.3]	10.8 [10.1, 11.4]	0.724
Low birth weight < 2.5 kg	41 (6.46)	58 (8.38)	0.183
Low birth weight ≤ 2.5 kg	93 (14.7)	118 (17.1)	0.231
Birthweight (kg)	3.01 [2.97, 3.05]	3.00 [2.96, 3.04]	0.605
N	635	692	
Female	308 (48.9)	351 (51.3)	0.379
Infant born during the rainy season (March - Sept)	428 (67.9)	465 (68.0)	0.986
N	630	684	
Household-level			
Age of head	38.5 [36.9, 40.2]	40.6 [39.4, 41.8]	0.010
Household size	6.16 [5.88, 6.44]	6.78 [6.46, 7.09]	<0.001
Head is married	597 (94.0)	647 (93.5)	0.697
Head is female	67 (10.6)	95 (13.7)	0.077
Head has no formal schooling	462 (72.8)	547 (79.1)	0.007
District			
East Mamprusi	271 (42.7)	253 (36.6)	0.023
Karaga	35 (5.51)	35 (5.06)	0.712
Yendi	57 (8.98)	53 (7.66)	0.385
Bongo	151 (23.8)	198 (28.6)	0.046
Garu-Tempane	121 (19.1)	153 (22.1)	0.170
N	635	692	

^a^ Generated using chi-square for dichotomous variables and bivariate linear regression models for continuous variables (treatment group as the independent variable).

Survey weights are applied to the sample analyzed in the table above to adjust for clustering of standard errors at the community-level. ANC, Antenatal care; CI, Confidence interval; PMT, score; proxy means test score. This descriptive table presents comparisons of maternal-, infant-, and household-level variables between comparison and treatment arms of the LEAP1000 program at baseline, irrespective of attrition by endline.

MI of missing birthweight yielded a sample of 3,258 infants aged 0–36 months after first imputing age in months. The differences between the samples with and without birthweight are shown in Supplementary Table S1 and demonstrate that there are systematic differences between these samples that we attempt to overcome with MI.

The impacts of LEAP1000 on continuous multiple imputed birthweight and LBW are presented in Table 2. Household size was removed from the adjusted models due to multicollinearity. At baseline, the average birthweights were 3.07 and 3.09 kg for the treatment and comparison groups, respectively, whereas at endline, the treatment group mean birthweight was 3.06 kg while the comparison mean birth weight decreased to 2.99 kg. The overall DID model (Table 2) shows that infants born to women in treatment households weighed on average 94 g more at birth and had approximately 4 percentage point (pp) lower LBW prevalence than infants born to women in comparison households (*p* < 0.001; Table 2, Panel A). For infants born the in dry season, LEAP1000 resulted in a 6.4 pp reduction in LBW (≤2.5 kg; *p* = 0.008), 4.1 pp reduction in LBW (<2.5 kg; *p* = 0.014), and a 109 g increase in birthweight (*p* = 0.003), on average (Table 2, Panel B). Among infants born in the rainy season, treatment infants weighed 79 g more, on average, than those in the comparison group (Table 2, Panel C; *p* = 0.029).

**TABLE 2:** Overall and season-specific impacts of LEAP1000 program on LBW and birthweight. Livelihood empowerment against poverty in the first 1,000 days of life impact evaluation, Ghana, 2015–2017.

A. Overall impacts^a^						
Dependent variable	Program impact [95% CI] *p*-value	Baseline treated mean	Baseline comparison mean	Endline treated mean	Endline comparison mean	
LBW (≤2.5 kg)	−0.038** [−0.077, −0.009] 0.014	0.112	0.103	0.122	0.148	
LBW (<2.5 kg)	−0.035** [−0.068, −0.010] 0.008	0.108	0.101	0.089	0.115	
Birthweight (kg)	0.094*** [0.045, 0.133] <0.001	3.069	3.087	3.056	2.987	
B. Dry season^b^						
LBW (≤2.5 kg)	−0.064** [−0.068, −0.010]0.008	0.111	0.104	0.105	0.165	
LBW (<2.5 kg)	−0.041** [−0.077, −0.008] 0.014	0.104	0.102	0.072	0.110	
Birthweight (kg)	0.109*** [0.035, 0.171] 0.003	3.074	3.090	3.073	2.977	
C. Rainy season^b^						
LBW (≤2.5 kg)	−0.018 [−0.074, 0.027] 0.358	0.113	0.102	0.135	0.138	
LBW (<2.5 kg)	−0.031 [−0.085, 0.010] 0.125	0.111	0.100	0.101	0.119	
Birthweight (kg)	0.079** [0.008, 0.143] 0.029	3.066	3.086	3.044	2.995	

^a^ Estimated using DID method.

^b^ Estimated using DDD method and lincom command in Stata.

Standard errors clustered at community-level. ***p-value < 1%; **p-value < 5%. LBW, low birth weight. Models are adjusted for female household head, age and schooling of household head, number of household members aged 0–17 years old, district of residence, month of birth, ANC from a skilled provider, and PMT score.

Table 3 presents the regression results of the LEAP1000 impact evaluation on birthweight using our complete-case sample (N = 1,567). We find no statistically significant impacts of LEAP1000 on birthweight among the overall complete-case sample, although there is a marginally significant increase in average birthweight (82 g; *p* = 0.094). Among infants born in the dry season, LEAP1000 led to an 18.5 pp reduction in LBW (≤2.5 kg; *p* = 0.003), a 9.6 pp reduction in LBW (<2.5 kg; *p* = 0.044), and a 207 g increase in average birthweight (*p* = 0.004), with no impacts on infants born in the rainy season. These complete case results should be interpreted with caution as we find numerous significant differences between the complete case and incomplete case analyses, especially pertaining to healthcare access and utilization (Supplementary Table S2). In both Tables 2, 3, the season-specific LEAP1000 impacts on LBW and birthweight have CIs that overlap and β_7_ (the coefficient for the DDD) was not significant for any outcome, indicating that there are non-significant differences in the impacts of LEAP1000 on infant birthweight and LBW in the dry and rainy seasons.

**TABLE 3:** Estimates of LEAP1000 impacts on birthweight and LBW among the complete case sample (N = 1,567 infants). Livelihood empowerment against poverty in the first 1,000 days of life impact evaluation, Ghana, 2015–2017.

A. Overall impacts^a^						
Dependent variable	Program impact [95% CI] *p*-value	Baseline treated mean	Baseline comparison mean	Endline treated mean	Endline comparison mean	
LBW (≤2.5 kg)	−0.056 [−0.138, 0.026] 0.179	0.170	0.135	0.141	0.163	
LBW (<2.5 kg)	−0.042 [−0.108, 0.025] 0.217	0.086	0.056	0.073	0.084	
Birthweight (kg)	0.082* [−0.014, 0.177] 0.094	3.004	3.020	3.021	2.958	
B. Dry season^b^						
LBW (≤2.5 kg)	−0.185*** [−0.306, −0.065] 0.003	0.204	0.102	0.111	0.185	
LBW (<2.5 kg)	−0.096** [−0.189, −0.002] 0.044	0.088	0.030	0.042	0.074	
Birthweight (kg)	0.207*** [0.069, 0.346] 0.004	2.965	3.077	3.047	2.946	
C. Rainy season^b^						
LBW (≤2.5 kg)	0.018 [−0.091, 0.128] 0.742	0.155	0.149	0.162	0.141	
LBW (<2.5 kg)	−0.013 [−0.118, 0.091] 0.801	0.085	0.068	0.095	0.094	
Birthweight (kg)	0.009 [−0.140, 0.158] 0.902	3.021	2.995	3.004	2.969	

^a^ Estimated using DID method.

^b^ Estimated using DDD method.

Notes: Standard errors clustered at community-level. * 10% significance ** 5% significance; *** 1% significance. Models are adjusted for female household head, age and schooling of household head, number of household members aged 0–17 years old, district of residence, month of birth, ANC from a skilled provider, and PMT score.

Table 4 presents the program impacts by the number of months of rainy season exposure *in utero* during the last 3 months of pregnancy among the complete-case sample. We find no significant program impacts on birth weight with increasing number of months in the rainy season *in utero* in the last 3 months of pregnancy.

**TABLE 4:** LEAP1000 program impacts on birthweight by number of months in the last 3 months of pregnancy in the rainy season (N = 1,454; among the complete-case sample). Livelihood empowerment against poverty in the first 1,000 days of life impact evaluation, Ghana, 2015–2017.

Dependent variable	Number of months in the rainy season		
	Program impact [95% CI] *p*-value		
	1	2	3
LBW (≤2.5 kg)	−0.01 [−0.239, 0.218] 0.927	−0.065 [−0.257,0.127] 0.505	−0.074 [−0.204, 0.056] 0.264
LBW (<2.5 kg)	−0.086 [−0.25, 0.077] 0.297	−0.113 [−0.311, 0.085] 0.260	0.002 [−0.097, 0.099] 0.976
Birthweight (kg)	−0.086 [−0.379, 0.208] 0.565	0.193 [−0.119, 0.506] 0.223	0.05 [−0.122, 0.223] 0.565

Notes: Standard error in parentheses. **p-value < 5%; Estimated using DDD method and *lincom* command in Stata. LBW, low birthweight.

## Discussion

This study found that Ghana LEAP1000 led to decreased LBW prevalence and increased average birthweight among live births overall and in the dry season, with increased average birthweight observed for infants born in the rainy season also. These findings confirmed our hypothesis that the LEAP1000 program would improve birthweight for infants born during the dry season. Due to the limited amount of the LEAP1000 cash transfer and the sheer burden of stressors present in the rainy season, we were not confident that the LEAP1000 program could help infants born in the rainy season overcome the great vulnerabilities associated with this season. This study adds to the extant literature of potential impacts of social protection programs on birthweight, with a particular and new focus on a rural Ghanaian population and the seasonality of program benefits in relation to vulnerabilities specific to maternal and child health.

Our research findings align with those of the scant, but promising, literature exploring cash transfer impacts on birthweight and LBW in LMICs. A recent review by Leroy and colleagues included four studies that examined cash transfer impacts on birthweight and showed meaningful increases in average birthweight of infants born to transfer beneficiaries (24). However, the overall quality of evidence and methodological approaches of these studies were ranked as low, and the authors suggest evaluations with more rigorous designs, providing the motivation for our study and approaches. Furthermore, none of the studies included in Leroy and colleagues’ review examined unconditional cash transfer programs and no studies were conducted in Africa. Mexico’s *Oportunidades* conditional cash transfer program has been associated with 102–127 g increases in birth weight, on average, and 4.4–4.6 percentage point decreases in LBW prevalence among infants born to participating women (40, 41). Amarante et. al. (2016) found that participation in Uruguay’s *Plan de Atención Nacional a la Emergerncia Social (PANES)* resulted in 1.9–2.5 percentage point reductions of LBW prevalence and a 31 g increase in average birth weight (42). A cluster-randomized controlled trial of the Participatory Learning and Action (PLA) program in Nepal found that women in the PLA plus food arm gave birth to infants 78 g heavier than women in the control arm (43). The findings in our study are comparable to those of the other studies reviewed by Leroy and colleagues. These studies report a range of program impacts on birthweight between 31 and 127 g and LBW reductions between 1.9 and 4.6 pp. Our findings fall in these ranges with estimated impacts on birthweight ranging from 79–207 g and reductions in LBW ranging from 3.5–9.6 pp.

No studies to date have examined the seasonal differences of cash transfer impacts on birth outcomes. There is, however, extensive evidence to suggest that the pathways relevant to our study impacts exhibit seasonality. A randomized controlled trial conducted to test the efficacy of a maternal dietary supplement on birthweight in the Gambia found significant increases in birthweight for the entire year, though the magnitude of effect was larger for the hungry (rainy) season than for the dry season and the effects of the intervention on LBW were similar across seasons (44). However, the seasonal variation in birthweight was not eliminated by supplementation in this study, suggesting that undernutrition or malnutrition are not solely responsible for seasonality of birth weight, giving credence to the importance of this study to explore other potential interventions to improve birthweight. Other studies suggest that the agricultural demands compounded by the rise in malarial infections during the rainy season may contribute to the seasonality of birthweight in SSA (45–50). Birthweight seasonality is also attributed to temperature exposures, which may directly affect pregnant women and impair fetal development, resulting in LBW (51).

The differences in the seasonality of intervention impacts on birthweight in our study compared to Ceesay and colleagues may be due to the direct provision of nutritional supplements in their study (44). However, a randomized controlled trial in Zimbabwe found no differential impacts of a micronutrient supplement on birthweight by season of birth (52). Furthermore, a qualitative study suggests that, while the rainy season is synonymous with hunger in many studies, focus group discussion participants in Uganda reported more food availability in the rainy season accompanied by increased physical labor, while in the dry season work conditions were considerably more difficult due to heat exposures (48). Our hypothesis of stronger and more significant impacts of the LEAP1000 program on infants born in the dry season was informed by the relatively small transfer value of the cash transfer, the heightened vulnerabilities of the rainy season including food insecurity and disease susceptibility, and literature that suggests rain exposure increases (rather than decreases) birthweight (53).

### Limitations

A notable limitation of this study is that the parallel trends assumption which is critical for validity of the DID model cannot be tested and we simply infer parallel trends by balance in outcomes across study groups at baseline. Forty percent of infants were not born in a health facility and 50% were not weighed at birth. Therefore, our small sample of 1,567 births is derived from 50% of all births, however, using MI to address this potential bias showed results similar to our complete case findings. However, these missing data approaches do not account for missing not at random mechanisms, which could result in invalid estimates. It is likely that infants born in facilities with low or very low birthweight were tended to for other pressing clinical procedures rather than being weighed immediately or at all, and this could be a potential mechanism for missing not at random, though we cannot test this (54–59). Birthweight reflects both preterm birth and intrauterine growth restriction and our data do not enable us to parse out these different contributors to LBW. We developed a dummy variable to indicate rainy versus dry season of birth for each infant, which is likely to misclassify the season of birth for some infants given the abrupt definition of the season variable that may not consider the variability of precipitation within the months. However, we did run sensitivity analyses to examine the potential misclassification resulting from this season indicator. While we did observe baseline imbalances in LBW across treatment arms for infants born in the dry season, the DID model controls for baseline differences between treatment groups, mitigating concern about these differences biasing our estimates. Moreover, we observed a change in the loading of birth frequency between baseline and endline suggesting a potential seasonal influence on fertility among this Ghanaian sample. Other studies on birthweight restrict samples to singletons, however, given our already limited sample size, we opted to include multiple births (3%) of birthweight observations, and this decision may have resulted in biased impact measures. Moreover, underlying LEAP1000 treatment impacts on infant viability, ultimately influencing infants’ live births, would obscure our ability to infer causation.

### Strengths

This is the first study to examine the seasonal impacts of an unconditional cash transfer program on birth weight in rural Ghana. We find that the LEAP1000 program leads to meaningful reductions in LBW prevalence among infants born in the dry season. Despite having missing birthweight data for 50% of our sample, we used multiple approaches and tested numerous assumptions related to missingness mechanisms and there is literature to suggest minimal bias is introduced for missing outcome data that are measured once per individual so long as predictors of missingness are included as covariates in models (60). Further research studying the seasonal impacts of social protection programs on pregnancy and birth outcomes in other settings could contribute to this body of knowledge. A methodological improvement to our study would be the utilization of Fourier series or other smooth functions for seasonality. Another improvement would include defining dry and rainy seasons at the local level by linking precipitation data for each community where the households are located given variations in precipitation patterns may exist for the Upper East and Northern regions. Further, understanding of the seasonal heterogeneity of outcomes that social protection programs seek to address would improve program implementation and population health.

## Conclusion

Based on our findings, we suggest that the provisions of LEAP1000 to pregnant and lactating women are not enough to overcome the many challenges women face, specifically during the rainy season, and suggest complementary programming be explored to improve the wellbeing of these mothers and their children. LEAP1000 is one of many cash transfer programs that has an objective of reducing stunting in the population. However, targeting of women during pregnancy or even pre-conception has not been a priority, which we consider to be a missed opportunity to intervene on adverse health and development outcomes during what has been considered to be the most critical periods.
