# Supplementary material for: Ghana’s Livelihood Empowerment Against Poverty (1000) Program Seasonally Impacts Birthweight: A Difference-in-Differences Analysis
Source: Int J Public Health. 2023 Feb 20;68:1605336. doi: 10.3389/ijph.2023.1605336 (PMC9986251; doi:10.3389/ijph.2023.1605336)
Supplement: Supplementary file 1 [file Table1.docx]

**Supplementary Table 1. Comparisons of characteristics by availability of birthweight**

| **A. Baseline (N=2,470)** | **Mean ± SD or N (%)** | |  |
| --- | --- | --- | --- |
|  | No birthweight available | Birthweight available | p-value* |
| Household |  |  |  |
| Household size | 6.90 ± 2.8 | 6.54 ± 2.46 | <0.001 |
| Number of household members 65 years or older | 0.24 ± 0.52 | 0.26 ± 0.52 | 0.352 |
| Household head is married | 1,116 (98) | 1,244 (94) | <0.001 |
| Household head is female | 61 (5) | 162 (12) | <0.001 |
| Age of household head | 38.8 ± 11.8 | 39.5 ± 13.4 | 0.156 |
| Education level of household head | 2.87 ± 7.79 | 5.16 ± 10.39 | <0.001 |
| Household head has no formal education | 978 (86) | 1,009 (76) | <0.001 |
| Household has no electricity | 820 (72) | 948 (71) | 0.868 |
| Number of household members 5 years or younger | 2.20 ± 0.95 | 2.03 ± 0.90 | <0.001 |
| PMT score | 7.16 ± 0.08 | 7.15 ± 0.08 | 0.303 |
| Mother |  |  |  |
| Number of meals consumed per day | 0.36 ± 0.48 | 0.42 ± 0.49 | <0.001 |
| Singleton delivery | 1,113 (98) | 1,265 (96) | 0.002 |
| Attended ANC | 1,083 (95) | 1,314 (99) | <0.001 |
| Delivered in a health facility | 282 (25) | 1,226 (92) | <0.001 |
| Child |  |  |  |
| Child age (months) | 11.5 ± 8.93 | 10.7 ± 8.23 | 0.036 |
| Index child | 978 (86) | 1,168 (88) | 0.072 |
| District |  |  |  |
| East Mamprusi | 312 (27) | 524 (40) | <0.001 |
| Karaga | 391 (34) | 70 (5) | <0.001 |
| Yendi | 288 (25) | 110 (8) | <0.001 |
| Bongo | 37 (3) | 349 (26) | <0.001 |
| Garu-Tempane | 115 (10) | 274 (21) | <0.001 |
| *N* | 1,143 | 1,327 |  |

| **B. Endline (N=702)** |  |  |  |
| --- | --- | --- | --- |
| Household |  |  |  |
| Household size | 6.61 ± 2.57 | 6.34 ± 2.59 | 0.175 |
| Number of household members 65 years or older | 0.14 ± 0.40 | 0.26 ± 0.51 | <0.001 |
| Household head is married | 344 (99) | 341 (96) | 0.008 |
| Household head is female | 9 (3) | 30 (9) | 0.001 |
| Age of household head | 36.9 ± 10.2 | 37.9 ± 12.3 | 0.226 |
| Education level of household head | 2.54 ± 7.16 | 4.36 ± 8.71 | 0.003 |
| Household head has no formal education | 302 (87) | 272 (77) | <0.001 |
| Household has no electricity | 255 (74) | 260 (73) | 0.941 |
| Number of household members 5 years or younger | 1.99 ± 1.11 | 1.72 ± 1.06 | 0.001 |
| PMT score | 7.17 ± 0.08 | 7.16 ± 0.08 | 0.085 |
| Mother |  |  |  |
| Number of meals consumed per day | 0.32 ± 0.47 | 0.42 ± 0.49 | 0.006 |
| Singleton delivery (N=677) | 326 (98) | 337 (98) | 0.555 |
| Attended ANC | 335 (97) | 349 (98) | 0.138 |
| Delivered in a health facility | 76 (22) | 334 (94) | <0.001 |
| District |  |  |  |
| East Mamprusi | 114 (33) | 147 (41) | 0.019 |
| Karaga | 102 (29) | 17 (5) | <0.001 |
| Yendi | 113 (33) | 28 (8) | <0.001 |
| Bongo | 9 (3) | 84 (24) | <0.001 |
| Garu-Tempane | 9 (3) | 79 (22) | <0.001 |
| *N* | 347 | 355 |  |
