# Supplementary material for: Ghana’s Livelihood Empowerment Against Poverty (1000) Program Seasonally Impacts Birthweight: A Difference-in-Differences Analysis
Source: Int J Public Health. 2023 Feb 20;68:1605336. doi: 10.3389/ijph.2023.1605336 (PMC9986251; doi:10.3389/ijph.2023.1605336)
Supplement: Supplementary file 2 [file Table2.docx]

**Supplementary Table 2. Comparison of characteristics between complete and non-complete case samples**

|  | Complete case | Non-complete case | p-value* |  |
| --- | --- | --- | --- | --- |
| Household-level |  |  |  |  |
| Household size | 6.53 | 6.87 | <0.001 |  |
| Household head married | 0.94 | 0.98 | <0.001 |  |
| Female household head | 0.11 | 0.04 | <0.001 |  |
| Age of household head | | 39.01 | 38.22 | 0.068 |
| Education of household head | 5.06 | 2.83 | <0.001 |  |
| Household head has no formal education | 0.76 | 0.86 | <0.001 |  |
| Household has no electricity | 0.71 | 0.71 | 0.952 |  |
| Number of children in the household aged 5 years or younger | 1.96 | 2.14 | <0.001 |  |
| PMT score | 7.15 | 7.16 | 0.058 |  |
| District |  |  |  |  |
| East Mamprusi | 0.41 | 0.32 | <0.001 |  |
| Karaga | 0.05 | 0.32 | <0.001 |  |
| Yendi | 0.08 | 0.26 | <0.001 |  |
| Bongo | 0.26 | 0.03 | <0.001 |  |
| Garu-Tempane | 0.20 | 0.08 | <0.001 |  |
| Mother-level |  |  |  |  |
| Singleton births | 0.97 | 0.98 | 0.009 |  |
| ANC from a skilled health professional | 0.99 | 0.95 | <0.001 |  |
| Delivery in a health facility | 0.93 | 0.27 | <0.001 |  |
| N | 1,567 | 1,513 |  |  |

*Estimated using chi-square tests for dichotomous variables and Studentized t-tests for continuous variables.

ANC: Antenatal care; PMT: Proxy means test
