# Supplementary material for: Ghana’s Livelihood Empowerment Against Poverty (1000) Program Seasonally Impacts Birthweight: A Difference-in-Differences Analysis
Source: Int J Public Health. 2023 Feb 20;68:1605336. doi: 10.3389/ijph.2023.1605336 (PMC9986251; doi:10.3389/ijph.2023.1605336)
Supplement: Supplementary file 3 [file DataSheet1.docx]

**A)**

1. ****

**Supplementary Figure 1. Monthly distribution of average birth weight and 95% confidence intervals for A) births at baseline (N=1,212) and B) new births at endline, by treatment status (N=355).**
